# Nanotechnology: from In Vivo Imaging System to Controlled Drug Delivery

**DOI:** 10.1186/s11671-017-2249-8

**Published:** 2017-08-17

**Authors:** Maria Mir, Saba Ishtiaq, Samreen Rabia, Maryam Khatoon, Ahmad Zeb, Gul Majid Khan, Asim ur Rehman, Fakhar ud Din

**Affiliations:** Department of Pharmacy, Quaid-I-Azam University, Islamabad, Pakistan

**Keywords:** Nanotechnology, Nanocomposites, In vivo imaging, Drug delivery and pharmaceutical nanosystems

## Abstract

Science and technology have always been the vitals of human’s struggle, utilized exclusively for the development of novel tools and products, ranging from micro- to nanosize. Nanotechnology has gained significant attention due to its extensive applications in biomedicine, particularly related to bio imaging and drug delivery. Various nanodevices and nanomaterials have been developed for the diagnosis and treatment of different diseases. Herein, we have described two primary aspects of the nanomedicine, i.e., in vivo imaging and drug delivery, highlighting the recent advancements and future explorations. Tremendous advancements in the nanotechnology tools for the imaging, particularly of the cancer cells, have recently been observed. Nanoparticles offer a suitable medium to carryout molecular level modifications including the site-specific imaging and targeting. Invention of radionuclides, quantum dots, magnetic nanoparticles, and carbon nanotubes and use of gold nanoparticles in biosensors have revolutionized the field of imaging, resulting in easy understanding of the pathophysiology of disease, improved ability to diagnose and enhanced therapeutic delivery. This high specificity and selectivity of the nanomedicine is important, and thus, the recent advancements in this field need to be understood for a better today and a more prosperous future.

## Review

### Introduction

As a matter of fact, nanotechnology is making progress through all imperative fields of engineering and science, and scientists are revolutionizing all the industries and human lives by designing things capable of working on the smallest scale length, atom by atom [1]. Nanotechnology involves the study of eminently small structures. Nanotechnology can be defined comprehensively as the study, creation, design, synthesis, and implementation of functional materials, systems, and devices through controlling matter within the size range of 1–100 nm at the nanometer scale. Moreover, the manipulation of innovative phenomena and improved properties of matter at this nanometer scale, also referred as molecular nanotechnology, is a magical point on scale length where smallest man-made appliances encounter the molecules and atoms of the universe [2–4].

The early inception of the concept of nanotechnology and nanomedicine sprang from the discerning idea of Feynman that tiny nanorobots and related devices could be developed, fabricated, and introduced into the human body to repair cells at molecular level. Although later in the 1980s and 1990s, this innovative concept was advocated in the famous writings of Drexler [5, 6], and in 1990s and 2000s in the popular writings of Freitas [7, 8]. Feynman offered the first known proposal for a nanomedical procedure to cure heart disease. In general, miniaturization of medical tools will provide more accurate, controllable, reliable, versatile, cost-effective, and quick approaches for improved quality of human life [9]. In 2000, for the very first time, National Nanotechnology Initiative was launched; then from onwards, modeling of electronics and molecular structures of new materials, establishment of nanoscale photonic and electronic devices [10, 11], development of 3D networking, nanorobotics [12], and advent of multi-frequency force microscopy [13] have paved the way for emergence of molecular nanotechnology.

Nanoparticles are considered as the essential building blocks of nanotechnology. Presence of strong chemical bonds, extensive delocalization of valence electrons varying with size, and structural modifications in nanoparticles lead to different physical and chemical properties including melting points, optical properties, magnetic properties, specific heats, and surface reactivity. These ultrafine nanoparticles exhibit completely new and improved properties as compared to their bulk counterpart due to variation in specific characteristics such as size, distribution, and of the particles which give rise to larger surface area to volume ratio [14–16]. As the field of nanostructured materials has been evolved, many different labels and terminologies are being used including 3D nanoparticle, nanocrystals, nanofilms, nanotubes, nanowires, and quantum dots with promising potential of infinite number of properties [17]. Because of the variety of potential applications (including industrial and military), governments have invested billions of dollars in nanotechnology research. The USA has invested 3.7 billion dollars through its National Nanotechnology Initiative, and European Union has also subsidized 1.2 billion, and 750 million dollars were invested by Japan [18].

Today, nanotechnology is one of the most innovative, vanguard areas of scientific study, and it continues to progress at staggering rates [19]. Through advancement in nanotechnology, many state-of-the-art technologies became available for the drug delivery. Researchers have extensively investigated the potential of nanodevices for target specific and controlled delivery of various micro- and macromolecules including drugs, proteins, monoclonal antibodies, and DNA (deoxyribonucleic acid) in multifarious biomedical applications like cancer [20, 21], vaccination [22], dental [23], inflammatory [24], and other health disorders. It is therefore a need of the day to demonstrate efficient use of nanotechnology applications ranging from in-vivo imaging system to controlled drug delivery, to mark the current progress and get directions for impending research in medical fields.

### Pharmaceutical Nanosystems

Pharmaceutical nanotechnology can be classified into two main categories of nanotools, i.e., nanomaterials and nanodevices. Nanomaterials can be further categorized on the basis of three basic parameters including structure, dimension, and phase composition. Nanostructures are further classified into polymeric and non-polymeric structures including nanoparticles, micelles, dendrimers, drug conjugates, metallic nanoparticles, and quantum dots [25]. On the basis of their dimensions, nanomaterials are classified in four groups, i.e., zero, one, two, and three nanodimension materials. According to phase composition, these nanomaterials can be categorized in three groups. Nanodevices are subdivided in three groups, including microelectromechanical systems/nanoelectromechanical system (MEMS/NEMS), microarrays, and respirocytes. These structures and devices can be fabricated with a high degree of functional property for use in medicine to interact with cells at a molecular level, thus allowing an extent of integration between biological systems and latest technology that was not achievable previously [26]. Detailed classification of pharmaceutical nanotools is described with their examples in Table 1.

**Table 1:** Pharmaceutical nanosystems (classification of nanotools)

Classification			Sub types	Examples	Structures	Applications	References
Pharmaceutical nanosystems	Nanomaterials	On basis of structure	Polymeric	Drug conjugates		• Deliver cytotoxic agents • Provide controlled release • Increase potency, tolerability and activity of drugs	[130]
				Micelles		• Amphiphilic block copolymers • Extremely small structure • Increase aqueous solubility of drugs	[131]
				Dendrimers		• Photodynamic therapy, boron neutron capture therapy • Potent anticancer agents	[132, 133]
			Nonpolymeric	Quantum dots		• Luminescent nanoprobes • Improved efficacy and bioavailability • Reduced side effects	[134]
				Carbon nanotubes		• Increase drug solubility and stability • Targeted drug delivery • Combination therapy	[135]
				Metallic nanoparticles		• Contrast agents • Provide controlled, targeted delivery	[136]
				Silica nanoparticles		• Improved pharmacokinetic profile • Enhanced bioavailability • Cornell dots	[137]
		Dimension wise	Zero-nanodimension	Spheres, clusters (fullerene)		• Production of nanoparticles • Functionalization of nanoparticles by dendritic structures	[138]
			One-nanodimension	Fibers, wires, rods		• Increase stability • Use in nanodevices, fibrils of nanodimensions, fabrication of polymer nanocomposites	[139, 140]
			Two-nanodimension	Films, plates, networks		• Used in sensing, electronics and optoelectronics	[141]
			Three-nanodimension	Tri and tetra pods, nanocombs		• Used in separation, catalytic, biomedical and heat transfer	[142]
		Phase composition wise	Single phase solids	Amorphous particles and layers		• Increase drug solubility • Increase the shelf life of drugs	[143]
			Multi-phase solids	Matrix composites		• Long term, repeated, on demand delivery of drugs for pain, chemotherapy, and insulin	[144]
			Multi-phase system	Colloids, ferro fluids		• Diagnosis and drug targeting • Deliver vaccines, toxoids, anticancer, gene and anti HIV drugs	[145]
	Nanodevices	NEMS/MEMS			• Microscopic devices with length more than 100 nm but less than 1 mm, possess combined electrical and mechanical components • Used for optical activities, electronic or biological applications and micro machines		[146]
		Microarrays			• Mapping of biological pathways, analysis of bio molecular interactions, assay development for compound screening, delivery of protein and peptides		[147]
		Respirocytes			• Artificial nanospherical robotic erythrocytes with internal pressure 1000 atm of combined oxygen and carbon dioxide • Preserve living tissues, treat anemia, asphyxia, and other respiratory problems		[148]

### Manufacturing Approaches

Nanosizing technologies have achieved great importance for the formulation of poorly water soluble drugs. By reducing the particle size to nanoscale range, the dissolution rate and bioavailability increase because of the increase in surface area, according to the Noyes-Whitney equation [27]. Approaches used for the manufacturing materials are categorized into bottom up techniques, top down techniques, and the combination of bottom up and top down techniques. Bottom up techniques involve built up of molecules. Some of the techniques that follow bottom up approach for manufacturing of nanoscale materials include liquid phase techniques based on inverse micelles, chemical vapor deposition (CVD), sol-gel processing, and molecular self-assembly. The components produced by bottom up are significantly stronger than the macroscale components because of the covalent forces that hold them together. In top down techniques, materials are micronized by cutting, carving, and molding for manufacturing of nanomaterials. Examples include milling, physical vapor deposition, hydrodermal technique electroplating, and nanolithography [28]. Different manufacturing approaches with their respective types are described in Table 2.

**Table 2:** Different approaches for manufacturing of various nanomaterials with their respective types

Approach	Subtypes	Principle	Example of drug	Nanostructure/nanodevice	References
Nanoprecipitation-dependent techniques	Anti-solvent precipitation	Supersaturation in which dissolution of a lipophilic drug in organic solvent followed by in anti-solvent (water). It leads to the nucleation of drug followed by precipitation of particles.	Itraconazole	Amorphous nanoparticles (<250 nm)	[149] [150]
			Curcumin	Nanoparticles	[151]
	Flash nanoprecipitation	Dissolution of a hydrophobic drug and amphiphilic copolymers in a water miscible organic solvent. Then, the organic solvent is mixed with an anti-solvent (water). High supersaturation level is achieved that triggers nanoprecipitaion.	Curcumin	Nanoparticles (40 nm)	[152]
			AIE (aggregation-induced emission) active dye of EDP	Fluorescent nanoparticles (20–60 nm)	[153]
			Doxorubicin	Nanoparticles (<100 nm)	[154]
	Sono precipitation	Crystallization by ultrasonic waves	Fenofibrate	Nanocrystals	[155]
			Felodipine	Nanosuspension	[156].
			Herceptin (HCT)-functionalized paclitaxel	Nanocrystals	[157]
			Lovastatin	Rod shaped nanocrystals	[158]
	High gravity controlled precipitation	High gravity conditions are maintained for precipitation by passing solution across rotating bed packing.	Hydroxyapatite (nHAP)	Nanoparticles (1.9–14.2 nm)	[159]
Milling-dependent techniques	Wet milling technique	Attrition is involved in which microsized particles are commuted by milling beads in a milling chamber to obtain nanosized particles (usually smaller than 400 nm).	Griseofulvin and Indomethacin	Nanoparticles (<100 nm)	[160]
			Itraconazole adipic acid	Nanocrystals	[161]
			Repaglinide	Nanocrystals	[162]
	Salt-assisted milling	Milling along with salts like NaCl with steel balls to produce nanosized particles. NaCl is incorporated in milling medium to prevent degradation and aggregation of nanoparticles [28].	Nanodiamond aggregates (50–1000 nm)	Nanodiamond colloids (5–10 nm)	[163]
	Co-grinding	Grinding of APIs with specific additives to produce nanosized particles [164].	Ibuprofen–glucosamine HCl	Co-ground particles	[165]
			Piroxicam	Cryogenic co-ground solid dispersions	[166]
High-pressure homogenization		Milling of suspended drug particles under high pressure by using homogenizer.	Myricetin	Nanosuspension	[167]
			α-chitin	Nanofibers (<100 nm)	[168]
Spraying-dependent techniques	Spray drying	Dispersion or liquids are transformed into solid powdered form upon spraying into drying medium at high temperature [169].	Cyclosporine A	Nanoparticles (317 to 681 nm)	[170]
	Electrospraying	Strong electric field is applied to atomize a liquid into fine dispersed particles at normal pressure and ambient temperature and without use of surfactants.	Piroxicam	Nanospheres	[171]
Supercritical fluid technology	RESS (Rapid expansion in supercritical solution)	Drug is solubilized in a supercritical fluid and the solution is then expanded in a low-pressure area through a nozzle. The drug becomes insoluble in low pressure gas and then supersaturation occurs and this leads to the production of micro and nanosized particles.	Olanzapine	Nanoparticles (150–350 nm)	[172]
	RESS-SC (Rapid expansion of supercritical solution with solid co-solvent)	In this technique, supercritical fluid, i.e., CO2, is saturated with several solid co solvents [173].	Theophylline	Nanoparticles (mean size: 85 nm)	[173]
	SAS (supercritical anti-solvent)	In this technique, precipitation of drug occurs upon its dissolution in an organic solvent, due to antisolvent effect.	Polyvinylpyrrolidone (PVP)–folic acid (FA)	Microspheres	[174]
			BSA (bovine serum albumin)	Nanoparticles (60 nm ± 10 nm)	[174]
	SAA (supercritical-assisted atomization)	The organic solution and supercritical carbon dioxide (SC-CO2) are mixed; they form an expanded liquid in a saturator. It is then atomized under some specific conditions results in the formation of nanodroplets which produce NPs by drying [175].	Rifampicin	PLLA nanoparticles (123 to 148 nm)	[176]
			Gentamycin sulfate	Microparticles (<2 μm)	[177]

### Biomedical Applications of Advanced Nanotechnology

#### Imaging

Tremendous advancements were reported during the last decade, using the nanotechnology tools for the imaging and therapy in research particularly targeting the cancer cells. Nanoparticles, with size 10–100 nm, offer a very suitable medium to carry out molecular level modifications such as the site-specific imaging and targeting in cancer cells [29]. The following section summarizes some recent advancement in the imaging techniques.

#### Radionuclide Imaging

Because of the inability of small molecules to be viewed with the noninvasive technique, the site-targeted contrast agents are employed to identify a selected biomarker that is impossible to be separated from the normal surrounding tissues [30]. The radionuclide imaging has been developed with the concept that the expressed protein is probed with a radiopharmaceutical or isotope-labeled agent or cell and is tracked further in vivo [31]. The positron emission tomography (PET) imaging is used in the cancer patients successfully to image the multidrug resistance through P-glycoprotein transport using 99 m tetrofosmin and sestamibi as the radiolabeled substrates for the P-glycoprotein [32, 33]. The mechanism of imaging is determined by the type of modality used for the imaging such as nanocarriers including liposomes [34], dendrimers [35], Bucky balls [36], and numerous polymers and copolymers [37]. They can be filled with the large number of imaging particles such as optically active compounds and radionuclides for the detection with imaging equipment. The BODIPY (boron dipyrromethane)-labeled jasplakinolide analogs have been used to visualize the long lived actin filaments inside the living cells [38, 39].

The enormous growth of nanotechnology is leading the research in the molecular imaging with many contrast agents. To obtain an appropriate imaging, the contrast agent selected should have longer half-life, low background signal, specific epitope binding, and enhanced contrast to noise enhancement. Large number of carrier availability is able to define more advancements in imaging with particular focus on the molecular and cellular mechanisms of the disease; this will create more opportunities for the rational development of imaging and drug delivery systems [30].

#### Quantum Dots

Semiconductor quantum dots are now used as a new class of fluorescent labels. These semiconductor nanocrystals are a promising tool for visualization of the biological cells owing to their easy surface chemistry, allowing biocompatibility and hereto conjugation with elongation of fluorescence time [29, 40]. The visualization properties of quantum dots (fluorescence wavelength) are strongly size dependent. The optical properties of quantum dots depend upon their structure as they are composed of an outer shell and a metallic core. For instance, grapheme quantum dots (GQD), a type of green fluorescence carbon nanomaterials, are made by cutting grapheme oxide solvothermally and are found to be dominating the visualization properties [41].

Quantum dot core is usually made up of cadmium selenide, cadmium sulfide, or cadmium telluride. The outer shell is fabricated on the core with high band gap energy in order to provide electrical insulation with preservation of fluorescence properties of quantum dots. The fine-tuned core and shells with different sizes and compositions with visualization properties of specific wavelength provide a large number of biomarkers [40]. Quantum dots are conjugated with different ligands in order to obtain specific binding to biological receptors. The tumor-targeting ligands are linked with amphiphilic polymer quantum dots and used to carry out the imaging studies of prostate cancer in mice [42]. Similarly, quantum dots offer significant advantages over the conventional dyes such as narrow bandwidth emission, higher photo stability, and extended absorption spectrum for the single excitation source. Moreover, the challenge of hydrophobicity in quantum dots has been overcome by making them water soluble. An example of the aqueous quantum dots with long retention time in biological fluids is the development of highly fluorescent metal sulfide (MS) quantum dots fabricated with thiol-containing charged groups [43]. Furthermore, the unique fluorescence properties of quantum dots made them suitable imaging tools for the cancer cells [42]. Quantum dots linked with A10 RNA aptamer conjugated with doxorubicin (QD-Apt-Dox) is the example of targeted cancer cell imaging [44]. However, increased toxicity of quantum dots has been observed due to the incorporation of heavy metals, resulting in their limited use for the in vivo imaging. Nevertheless, recent approaches focus on the reduction in toxicity and the enhancement of biocompatibility of quantum dots to the body cells. It is also worth to mention that quantum dots with the diameter less than 5.5 nm are rapidly and efficiently excreted from the urine resulting in reduced toxicity. This phenomenon was exhibited by the synthesis of cadmium free, CulnS_2_/ZnS (copper indium sulfide/ zinc sulfide) as the core and shell of the quantum dots, which resulted in enhanced stability in the living cells for lymph node imaging with a clear reduction in acute local toxicity [45, 46].

#### Biosensors

One of the greatest achievements in nanomaterials since last few years is the development of biosensors. Biosensors are the devices that contain the biological sensing element that is either connected or integrated in the transducer. Biosensor exhibits their action by recognition of specific molecules in the body on the basis of their structure including antibody antigen, enzyme substrate, and receptor hormone. The two major properties of biosensor including their specificity and selectivity are dependent upon this recognition system. These basic properties of the biosensors are most importantly used for the concentration that is proportional to the signals [47–49].

In order to produce the biosensor with high efficiency, the substrate selected for the sensing material dispersion is prerequisite. Different types of nanomaterial including quantum dots [50], magnetic nanoparticles [51], carbon nanotubes (CNTs) [52], and gold nanoparticles (GNPs) [53] are applied to the biosensors. The distinctive chemical, physical, magnetic, optical, and mechanical properties of nanomaterial lead to their increased specificity and sensitivity for detection. Biosensors containing GNPs have offered a compatible environment for the biomolecules that has increased the immobilized biomolecules concentration on the surface of electrode. It has resulted in enhanced sensitivity of the biosensors [54, 55]. The most widely used electrode surfaces within the biosensors are the glassy carbon electrode (GCE), which are modified from GNPs. Moreover, they have shown best sensitivity as well as electrochemical stability. In this regards, methylene blue (MB) and GNPs are easily assembled and modified through layer by layer (LBL) technique in the form of films on GCE, in order to detect the concentration of human chorionic gonadotrophin (HCG) [56]. Owing to the large surface area contained by the nanoparticles in order to load anti-HCG, these immunosensors have their potential to be used for detecting the concentrations of HCG in the human blood or urine samples. Similarly, CNTs have found great applications in biomedical engineering, bio-analysis, bio-sensing, and nanoelectronics [57–59]. Moreover, multi-walled carbon nanotubes (MWNT) in the form of bio-nanocomposite layers of polymers have the potential to be used for the DNA detection [60]. Furthermore, magnetic nanoparticles have also found wide applications because of their magnetic properties, including magnetic resonance imaging (MRI) contrast agent [61], hyperthermia [62], immunoassay [63], tissue repair [64], cell separation [65], GMR-sensor [66], and drug or gene delivery [67].

Likewise, a new type of magnetic chitosan microspheres (MCMS) has also been produced by simply using chitosan and carbon-coated magnetic nanoparticles [68]. In this study, hemoglobin was also immobilized successfully on the MCMS modified GCE surface by using glutaraldehyde as the crosslinking agent. Another important application of biosensors is in the optical technology, which includes the detection of various kinds of DNA oligonucleotides by using SsDNA–CNT probes as the biosensors [69]. Similarly, liposome-based biosensors have also gained considerable attention as they have been used in the monitoring of the organophosphorus pesticides, including paraoxon and dichlorvos on the minimum levels [70].

#### Magnetic Nanoparticles

Magnetic nanoparticles (MNPs) provide exclusive magnetic properties as they have the ability to work at the molecular or cellular level of the biological interactions, which make them the best compounds as contrast agents in MRI and as carriers in drug delivery. The recent advancements in nanotechnology have gained attention as it helped in the modification of the properties and features of MNPs for the biomedical applications. In this respect, the liver tumor and metastasis imaging via RES-mediated uptake of superparamagnetic iron oxides (SPIOs) has been shown to be capable of the differentiation of the lesions that are as small as only 2–3 mm [70, 71]. Moreover, these ultra-small supermagnetic iron oxides (USPIOs) are also very effective in the imaging of the metastasis of the lymph nodes with only 5 to 10 mm of diameter [72]. Furthermore, importance of this noninvasive approach has also been shown in the detection of the lymphatic dissemination as it is considered an important part in the staging as well as in identifying the treatment approaches for the breast colon and prostate cancers [73].

### Drug Delivery

Nanotechnology is an attractive tool for disciplines ranging from materials science to biomedicine because of their different physical, optical, and electronic characteristics. The most effective research areas of nanotechnology are nanomedicine that applies nanotechnology principles for the treatment, prevention, and diagnosis of diseases. Moreover, many products of nanomedicine have been marketed due to the surge in nanomedicine research during the past few decades, around the globe. Currently, nanomedicine is influenced by drug delivery systems, accounting for more than 75% of the total sales [74]. In this regards, nanoparticle-based drug delivery platforms have gain the trust of scientists for being the most appropriate vehicles in addressing the pharmacokinetic drawbacks associated with conventional drug formulations [75]. Hence, various nanoforms have been attempted as drug delivery systems such as liposomes, solid lipid nanoparticles, dendrimers, and solid metal-containing NPs, to enhance the therapeutic efficacy of drugs [76, 77]. Some of the major fields of interest are discussed below.

#### Ophthalmology

Drug delivery through the ophthalmic route is highly attractive yet challenging for the pharmaceutical scientists. The eye is a tiny intricate organ with multi-compartments. Its biochemistry, physiology, and anatomy have made it most impermeable to the xenobiotic. Common conditions that demand ocular administration contain the eye infections such as, conjunctivitis along with the corneal disorders like glaucoma. The most common drug classes used in the ocular delivery include mydriatics or cycloplegics miotics, anti-infective, anti-inflammatory, diagnostics, and surgical adjuvants. For the small ocular irregularity, gene therapy is required too, and a large amount of work is being conducted within this area. Nanocarrier supported approaches have got attention of the scientists for their suitability and specificity. It has been reported that particulate delivery system such as microspheres and nanoparticles and vesicular carriers like liposomes, niosomes, pharmacosomes, and discomes improved the pharmacokinetic and pharmacodynamics properties of various types of drug molecules [76]. Many novel controlled drug delivery systems have been emerged including hydrogels, muco-adhesive polymers, microemulsions, dendrimers, iontophoretic drug delivery, siRNA-based approaches, stem cells technology, non-viral gene therapy, and laser therapy with the sclera plugs [78]. Different systems for drug delivery are costumed for the delivery of drug through the ocular route. The chief goal of all the drug delivery systems is to improve the residence period, enhance the corneal permeability, and liberate the drug at posterior chamber of eye, leading to increased bioavailability and improved patient compliance [79].

Abrego et al. prepared PLGA (poly lactic co-glycolic acid) nanoparticles of pranoprofen for ophthalmic delivery in the form of hydrogel. This hydrogel formulation have suitable rheological and physicochemical properties for the ocular delivery of pranoprofen with improved biopharmaceutical outline of the drug. Moreover, it intensified the local anti-inflammatory and analgesic results of the drug, resulting in improved patient’s compliance [80]. In another study, cefuroxim loaded nanoparticles of chitosan were developed using a double crosslinking in double emulsion technique. The inference point out chitosan-gelatin particles as potently practical candidates for DD at intraocular level [81]. Moreover, diclofenac loaded N-trimethyl chitosan nanoparticles (DC-TMCNs) were developed for ophthalmic use to improve ocular bioavailability of the drug [82]. Furthermore, nanosized supramolecular assemblies of chitosan-based dexamethasone phosphate have been developed for improved pre-corneal drug residence time due to its muco-adhesive characteristics. These nanoparticles interact strongly with both ocular surface and drug and protect the drug from metabolic degradation leading to extended pre-corneal residence [83]. Glaucoma, an ophthalmic disease, was treated with brimonidine-based loaded sustained release solid lipid nanoparticles using glyceryl monostearate as solid lipid [84, 85]. Similarly, daptomycin-loaded chitosan-coated alginate (CS-ALG) nanoparticles were developed with a suitable size for ocular applications and high encapsulation efficiency (up to 92%). This study revealed that daptomycin nanocarrier system could be used in future to deliver this antibiotic directly into the eye, in order to act as a prospective therapy against bacterial endophthalmitis and as an efficient alternative to chitosan nanoparticles [86].

One of the major causes of short- and long-term failure of grafts in the corneal transplantation is the immunologic graft rejection. For this purpose, PLGA-based biodegradable nanoparticle system of dexamethasone sodium phosphate (DSP) was prepared, resulting in the sustained release of the corticosteroids in order to prevent the rejection of corneal graft [87]. Moreover, MePEG-PCL (polyethylene glycol-poly caprolactone) nanoparticles of curcumin were reported, and they showed increased efficiency, enhanced retention of curcumin in the cornea, and significant improvement in prevention of the corneal neovascularization over free curcumin [88]. Likewise, silver nanoparticle-infused tissue adhesive (2-octyl cyanoacrylate) were developed with enhanced mechanical strength and antibacterial efficacy. These doped adhesive (silver nanoparticles) supported the use of tissue adhesives as a viable supplement or alternative to sutures [89].

#### Pulmonology

Lung diseases probably asthma, chronic obstructive pulmonary disease (COPD), and lung cancer have a high occurrence and are often life threatening. For instance, it is described that COPD is the fourth major cause of death, and lung carcinoma is the most prevailing cause of cancer deaths worldwide. Nanoparticles are scrutinized as a choice to improve therapy of these severe diseases [90]. Various drug-laden nanoparticles have been utilized for their local and systemic effects in the treatment of lung diseases. Delivery of curative agents to the place of action for lung diseases may permit for effective treatment of chronic lung infections, lung cancers, tuberculosis, and other respiratory pathologies [91]. The nanocarriers used for this purpose include liposomes, lipid- or polymer-based micelles, dendrimers, and polymeric NPs [92]. Polymeric NPs are of prenominal interest, as the polymers can be co-polymerized, surface modified, or bio-conjugated for ameliorate targeting capacity and distribution of the encapsulated agents. The generally used nanocarriers in pulmonary drug delivery contain natural polymers such as gelatin, chitosan, and alginate and synthetic polymers like poloxamer, PLGA, and PEG [93].

**Fig. 1:**
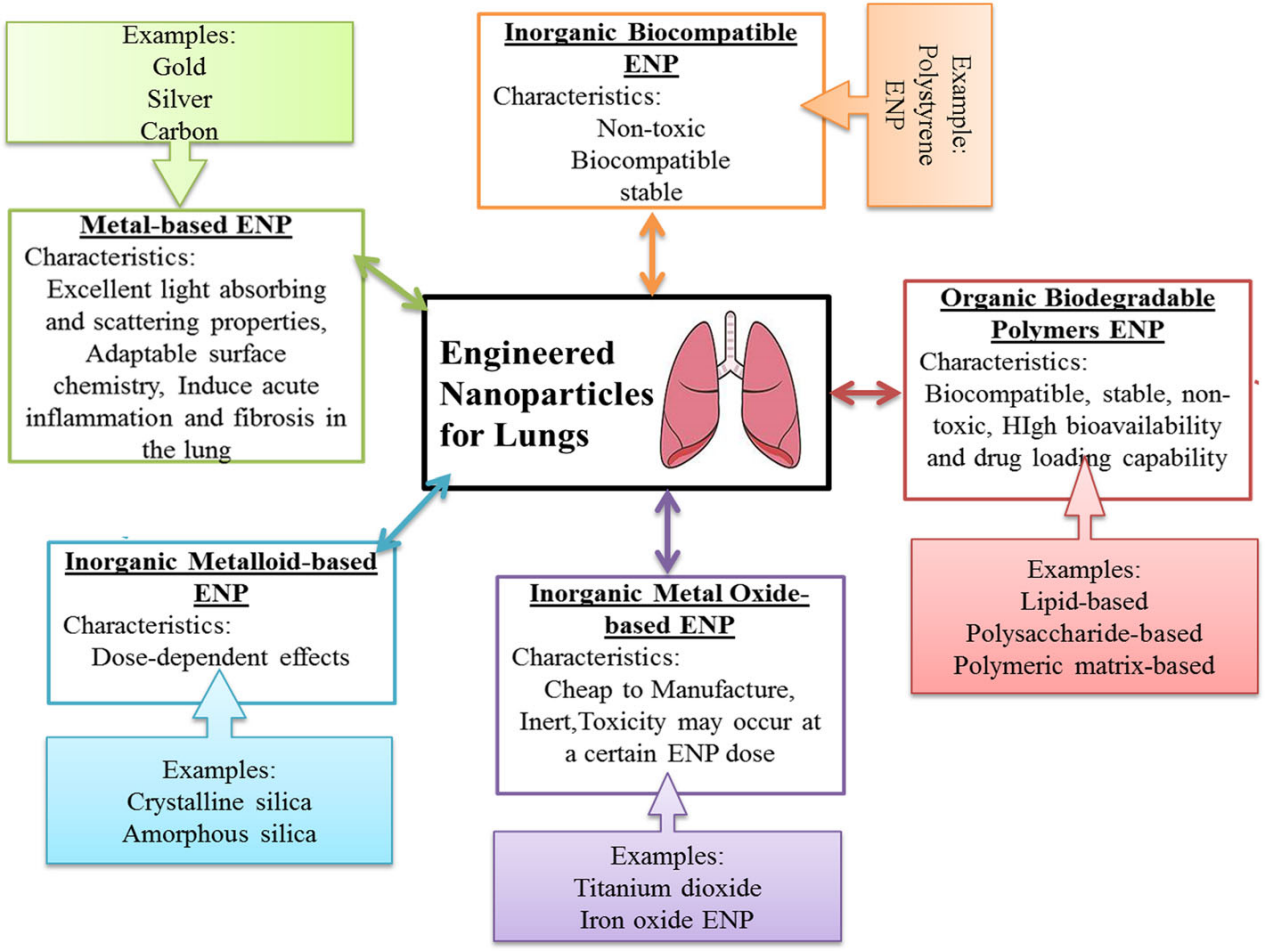
Properties and efficacious effects of ENPs on lungs

It was observed that PLGA NPs exhibit the most convenient set of characteristics as carriers for pulmonary protein/DNA delivery while gelatin NPs are an agreeable reciprocal choice [94]. Similarly, anisotropic or Janus particles of doxorubicin and curcumin were formulated to cargo the anticancer drugs for the treatment of lung cancer through inhalation. The particles were formulated by using the biocompatible and biodegradable materials binary mixtures. These particles did not exhibit geno- and cytotoxic consequence. The cancer cells internalize these Janus particles and massed them in the nucleus and cytoplasm leading to prolonged retention. Moreover, polyamidoamine (PAMAM) dendrimers were evaluated as nanocarriers for pulmonary delivery of the model weakly soluble anti-asthma pharmaceutical beclometasone dipropionate (BDP) using G3, G4 and G4 [12] dendrimers. This study showed that BDP-dendrimers have potential for pulmonary inhalation using air-jet and vibrating-mesh nebulizers. Furthermore, it was observed that the aerosol characteristics were influenced by nebulizer design rather than dendrimers generation [95]. Additionally, engineered nanoparticles (ENP), composed of inorganic metals, metal oxides, metalloids, organic biodegradable, and inorganic biocompatible polymers were used efficiently as carriers for the vaccine and drug delivery and for the management of a variety of lung diseases. Properties and efficacious effects of ENPs on lungs are represented in Fig. 1. Inorganic ENP (silver, gold, and carbon ENP), metal oxides ENP (iron oxide, zinc oxides, and titanium dioxide), and organic ENP (Lipid-based, polysaccharide-based, polymer matrix-based) were developed and evaluated for pulmonary immune hemostasis. As well as being relatively secure carriers, modern studies indicated ENP cable of supervening beneficial outcomes with anti-inflammatory properties (e.g., silver and polystyrene) and imprinting of the lung which present the maintenance of immune homeostasis (e.g., polystyrene). Further knowing of the mechanisms may help in better understanding the useful effects of ENP on pulmonary immune homeostasis and/or management of inflammatory lung disease [96].

It is important to state that functionalized cationic lipo-polyamine (Star: Star-mPEG-550) have been recently developed for the siRNA (short interference RNA) in vivo delivery to the pulmonary vascular cells. This balanced lipid formulation intensify the siRNA retention in the lungs of mouse and accomplished significant disassemble of the target gene. The results were found useful and with reduced toxicity of miRNA-145 inhibitor delivery to the lung by using the functionalized cationic lipopolyamine nanoparticles to recruit the pulmonary arteriopathy and rectify function of heart within rats with intense pulmonary arterial hypertension (PAH) [97].

#### Cardiovascular System

Cardiovascular disease is the ailment that affects the cardiovascular system, vascular diseases of the brain and kidney, and peripheral arterial disorder. Despite of all advances in pharmacological and clinical management, heart failure is a foremost reason of morbidity worldwide. Many novel therapeutic strategies, embody cell transplantation, gene delivery or therapy, and cytokines or other small molecules, have been studied to treat heart failure [98]. An inadequate number of people are affected in developing countries; over 80% of deaths due to cardiovascular disorder take place in underdeveloped countries and occur almost evenly in male and females [99]. Mathers et al. in 2008 estimated that there are 9.4 million deaths each year [100]. This concludes 45% of deaths caused by coronary heart disease and 51% of deaths due to heart strokes [101]. There are many distinct types of drug delivery vehicles, like polymeric micelles, liposomes, dendrimers, lipoprotein-supported pharmaceutical carriers, and nanoparticle drug carriers.

Chitosan-based liposomes of sirolimus having ≥83% entrapment efficiency were developed for the treatment of restenosis and have been proved a novel platform for efficient targeted delivery [102]. Similarly, bile salt-enriched niosomes of carvedilol with 85% entrapment efficiency have resulted in enhanced bioavailability of drug, and thus, better therapeutic effect [103] was obtained. Inhibition of restenosis in balloon-injured carotid artery is achieved in rats by developing PLGA-based nanoparticles encapsulating AGL 2043 and AG1295, selective blockers of platelet-derived growth factors (PDGF) receptors [104]. Angiogenic therapy of myocardial ischemia with vascular endothelial growth factor (VEGF) is a favorable approach to overcome hypoxia and its sequel effects. Polymeric particles loaded with VEGF have been proved a promising system for delivery of cytokines to rat myocardial ischemic model. This approach could be further explored for clinical studies [105]. Coenzyme Q10 (CoQ10) owing to its role in mitochondrial electron transport chain appears to be a reliable candidate to treat myocardial ischemia (MI) but its poor biopharmaceutical characteristics needed to be addressed by developing promising delivery approaches. Polymeric nanoparticles were developed to encapsulate CoQ10 to overcome its poor pharmaceutical properties and administered to MI-induced rats. Cardiac function was analyzed by determining ejection fraction before and after 3 months of therapy. Results showed significant betterment in the ejection fraction after 3 months [106].

#### Oncology

Cancer is a prime cause of mortality around the globe. The World Health Organization determines that 84 million people die of cancer between 2005 and 2015. The eventual target of cancer therapeutics is to increase the life span and the quality of life of the patient by minimizing the systemic toxicity of chemotherapy [107]. Chemotherapeutic agents have widely been studied in oncology for the past 25 years, but their tumor specificity is unsatisfactory and therefore exhibit dose-dependent toxicity. To overcome this limitation, recent interest has been centered on developing nanoscale delivery carriers that can be targeted directly to the cancer cell, deliver the drug at a controlled rate, and optimize the therapeutic efficacy [108, 109]. Passive and active targeting is used to deliver the drug at its tumor site. The passive phenomenon called the “enhanced permeability and retention (EPR) effect,” discovered by Matsumura and Maeda, is the dominated pathway used for chemotherapeutics [110, 111]. Active targeting is achieved by grafting ligand at the surface of nanocarriers that bind to receptors or stimuli-based carriers, e.g., dual reverse thermosensitive [112], photo-responsive [113], magnetic nanoparticles [114], and enzymatically activated pro-drugs [115]. Nanoparticles (NPs) can be conjugated with various smart therapeutic carriers like polymeric nanoparticles [116], micelles [117], liposomes [118], solid lipid nanoparticles (SLNs) [119], protein nanoparticles [120], viral nanoparticles [121], metallic nanoparticles [122], aptamers [123], dendrimers [124], and monoclonal antibody [125] to improve their efficacy and decrease the systemic toxicity. Table 3 summarizes the different approaches for drug deliveries which are widely studied to target the tumor with maximize therapeutic response and minimum toxicity.

**Table 3:** Nanomaterials and drug delivery approaches for tumor treatment

Nanomaterials	Delivery approaches	Advantages	References
Aptamer functionalized silica gold nanorods (60 nm)	Near-infrared light responsive drug delivery system	Biocompatibility, cancer cell recognition ability, and efficient intracellular drug release	[178]
Doxorubicin-loaded PEG diacrylate -Chitosan derivative-single-wall carbon nanotubes (CNT) (240 nm)	Near-infrared (NIR) light triggered drug delivery system	Enhanced cellular uptake and the faster drug release	[179]
(DOX)-loaded hollow mesoporous copper sulfide nanoparticles (HMCuS NPs) with iron oxide nanoparticles (IONPs) (124.5 ± 3.8 nm)	Near-infrared (NIR) light triggered drug delivery system	Minimized the adverse effects, enhanced photo thermal therapy effect	[180]
DOX-(HMCuSNPs) with hyaluronic acid (HA) (113.8 ± 6.9 nm)	Near infrared (NIR) light triggered drug delivery system	Facilitate intracellular tunable drug release, enhanced targeting and accumulation capacity in tumor site	[181]
α-Cyclodextrin and poly (ethylene glycol)-platinum dendrimer (1.9 ± 0.3 nm)	Near infrared (NIR) light-responsive supramolecular hydrogel	Enhanced release of drug, low toxicity	[182]
End-capped mesoporous silica nanoparticles (MSNs) (130 nm)	Redox-responsive nanoreservoirs	Excellent biocompatibility, cell-specific intracellular drug delivery, and cellular uptake properties	[183]
Transferrin (Tf)-(MSNs)-DOX (280 nm)	Redox-responsive drug delivery system	Biocompatible, enhanced intracellular accumulation, targeting capability	[184]
Amino- β –cyclodextrin- MSNs (203.3 nm)	Folate mediated and pH targeting	High intercellular release	[185]
DOX-thiolated poly(ethylene glycol)-biotin-DNA conjugated gold nanorod (GNR) (length of 50 ± 5 nm diameter of 14 ± 3 nm)	pH-and near infrared (NIR) radiation dual-stimuli triggered drug delivery	Increased potency (~67-fold), increased cell uptake, low drug efflux	[186]
Cytochrome C conjugated lactobionic acid (CytC–LA)- Doxorubicin (DOX)- MSNs (115.8 nm)	pH and redox dual-responsive drug delivery	Good biocompatibility, high efficiency, inhibits tumor growth with minimal toxic side effect.	[187]
Poly (propylene sulfide)-polyethylene glycol-serine-folic acid (PPS-mPEG-Ser-FA)- zinc phthalocyanine-doxurubicin micelle (80 nm)	Reactive oxygen species (ROS) sensitive drug delivery system	Minimal toxic side effects	[188]
Rituximab-conjugated doxorubicin- MSNs (40.7 ± 19.1 nm)	pH-sensitive controlled drug release system	Reduce systemic toxicity, improve the therapeutic efficacy	[189]
PEGylated-MoS 2 nanosheets (diameter 50 nm, thickness ∼2 nm)	Combined photothermal and chemotherapy targeting	Highly efficient loading	[190]
DOX-Gold nanorod-1-tetradecanol-MSNs (thickness 35 nm)	Photothermalablation and chemotherapy	Precise control over drug release, localized delivery with enhanced targeting	[191]
Fe_3_O_4_–azobis [*N*-(2-carboxyethyl)-2-methylpropionamidine](Azo)-Doxorubicin	Combined photothermal therapy and chemotherapy	Enhanced cell-killing effects, increased stability, low toxicity	[192]

Biodegradable poly (o-caprolactone) nanocarriers loaded with tamoxifen were developed for the management of estrogen receptor-specific breast cancer [126]. This study suggested that the nanoparticle preparations of selective estrogen receptor modulators deliver the drug in the specific estrogen receptor zone resulting in enhanced therapeutic efficacy. Similarly, a nanoconjugation of doxorubicin and cisplatin was developed by Chohen et al. [127], which have exhibited enhanced efficiency and reduced side effects of the loaded drugs in the treatment of localized progressive breast cancer. Likewise, chemotherapeutic drug oxaliplatin-loaded nanoparticulate micelles were prepared by Cabral et al. [128], with sustained release of loaded drug in the tumor microenvironment, resulted in enhanced antitumor effect [128]. Furthermore, SLN loaded-5-FU resulted in enhanced bioavailability and sustained release of the encapsulated anticancer drug, leading to enhanced antitumor effect [129].

## Conclusions

Nanotechnology is subjected to inordinate progress in various fronts especially to make innovations in healthcare. Target-selective drug delivery and approaches for molecular imaging are the areas of prime importance for research where nanotechnology is playing a progressive role. This review provides readers with a wide vision on novel ongoing potentialities of various nanotechnology-based approaches for imaging and delivery of therapeutics. In order to obtain effective drug delivery, nanotechnology-based imaging has enabled us to apprehend the interactions of nanomaterials with biological environment, targeting receptors, molecular mechanisms involved in pathophysiology of diseases, and has made the real time monitoring of therapeutic response possible. Development of analytical technologies to measure the size of particles in nanometer ranges, and advent of latest manufacturing approaches for nanomaterials, has resulted in establishment of more effective methods for delivery of therapeutics for the treatment of ophthalmological, pulmonary, cardiovascular diseases, and more importantly cancer therapy. These new drug therapies have already been shown to cause fewer side effects and be more effective than traditional therapies. Furthermore, the imaging techniques have enhanced the determination of tumor location in human bodies and their selective targeting. Altogether, this comparatively new and thriving data suggest that additional clinical and toxicity studies are required further on the “proof-of-concept” phase. Nanomedicine cost and manufacturing at larger scale is also a matter of concern that needs to be addressed. Notwithstanding, future of nanomedicines is propitious.

## Abbreviations

AIE: Aggregation-induced emission
BDP: Beclometasone dipropionate
BODIPY: Boron dipyrromethane
CNTs: Carbon nanotubes
COPD: Chronic obstructive pulmonary disease
CulnS_2_/ZnS: Copper indium sulfide/zinc sulfide quantum dots
CVD: Chemical vapor deposition
DNA: Deoxyribonucleic acid
ENPs: Engineered nanoparticles
EPR: Enhanced permeability and retention
GCE: Glassy carbon electrode
GNPs: Gold nanoparticles
GQD: Grapheme quantum dots
HCG: Human chorionic gonadotrophin
MEMS: Microelectromechanical systems
MI: Myocardial ischemia
MNPs: Magnetic nanoparticles
MSNs: Mesoporous silica nanoparticles
MWNT: Multi-walled carbon nanotubes
NEMS: Nanoelectromechanical system
PAH: Pulmonary arterial hypertension
PCL: Poly caprolactone
PDGF: Platelet-derived growth factors
PEG: Poly ethylene glycol
PET: Positron emission tomography
PLGA: Poly lactic-co-glycolic acid
ROS: Reactive oxygen species
SiRNA: Short interference RNA
SLNS: Solid lipid nanoparticles
SPIOs: Superparamagnetic iron oxides
VEGF: Vascular endothelial growth factor
